# A Day in the Life of Fish Larvae: Modeling Foraging and Growth Using Quirks

**DOI:** 10.1371/journal.pone.0098205

**Published:** 2014-06-05

**Authors:** Klaus B. Huebert, Myron A. Peck

**Affiliations:** Institute for Hydrobiology and Fisheries Science, University of Hamburg, Hamburg, Germany; Institut Maurice-Lamontagne, Canada

## Abstract

This article introduces “Quirks,” a generic, individual-based model synthesizing over 40 years of empirical and theoretical insights into the foraging behavior and growth physiology of marine fish larvae. In Quirks, different types of larvae are defined by a short list of their biological traits, and all foraging and growth processes (including the effects of key environmental factors) are modeled following one unified set of mechanistic rules. This approach facilitates ecologically meaningful comparisons between different species and environments. We applied Quirks to model young exogenously feeding larvae of four species: 5.5-mm European anchovy (*Engraulis encrasicolus*), 7-mm Atlantic cod (*Gadus morhua*), 13-mm Atlantic herring (*Clupea harengus*), and 7-mm European sprat (*Sprattus sprattus*). Modeled growth estimates explained the majority of variability among 53 published empirical growth estimates, and displayed very little bias: 0.65%±1.2% d^−1^ (mean ± standard error). Prey organisms of ∼67% the maximum ingestible prey length were optimal for all larval types, in terms of the expected ingestion per encounter. Nevertheless, the foraging rate integrated over all favorable prey sizes was highest when smaller organisms made up >95% of the prey biomass under the assumption of constant normalized size spectrum slopes. The overall effect of turbulence was consistently negative, because its detrimental influence on prey pursuit success exceeded its beneficial influence on prey encounter rate. Model sensitivity to endogenous traits and exogenous environmental factors was measured and is discussed in depth. Quirks is free software and open source code is provided.

## Introduction

It is of considerable ecological interest to estimate the environmental conditions that are necessary to support feeding and growth of marine fish larvae, and to predict how well theoretical or observed conditions match larval requirements. “Matches” can facilitate growth, enhance survival, and increase fish recruitment potential, while “mismatches” can prevent larvae from thriving, and limit both the short-term productivity and the long-term biogeographic distribution of fish stocks [1], [2]. While many studies have related larval growth to one or more environmental factors, our understanding of the interplay between variables such as temperature, turbulence, and prey concentration is still quite limited. Individual-based models (IBMs) synthesizing empirical and theoretical insights can be used to apply and test our cause-and-effect understanding of the essential physical, behavioral, and physiological processes governing growth [3]. Recently, there has been a dramatic increase in studies employing larval fish IBMs to these ends [4], [5]. Most of these studies have focused on one fish stock or species and have not provided the model source code. Our new model “Quirks” is free and open source software written in the R programming language [6] (Source Code S1, https://sourceforge.net/projects/larvalfishquirks/), easy to parameterize for different types of larvae, and, as we show here, performs well across multiple species and environments.

### Design Goals

Our main technical objective was to provide the scientific community with a free and useful comparative modeling tool for the study of larval fish foraging and growth. To this end, we tried to make Quirks as transparent (understandable without prior modeling experience), mechanistic (based on cause-effect understanding of biophysical principles), and generic (appropriate for different types of larvae and environments) as possible. Further, we aimed to synthesize published data and did not “tune” Quirks to perform well in the presented applications. Quirks was written in R, a free programming language and software environment commonly used for statistical data analysis, which is familiar to many scientists besides modelers [6]. The underlying equations were kept simple, while still mechanistically representing physiological processes and interactions between larvae and their biophysical environment. A trait-based modeling approach was chosen for simple parameterization and comparison of different larval types. Trait-based modeling has become a popular tool to investigate how marine phytoplankton [7] or zooplankton [8] are adapted to prevailing environmental conditions, and trait-based IBMs have previously been used to compare exogenous and endogenous factors influencing larval fish survival [9]. The standard dynamic energy budget (DEB) model [10] is another bioenergetic model that can be considered trait-based, and has been applied to study, for example, larval fish stage duration [11]. However, DEB traits are not particularly transparent (some cannot or have not been measured in fish larvae, and are parameterized by fitting model output to empirical growth curves). Further, standard DEB theory does not mechanistically represent foraging behavior. In summary, Quirks uniquely represents larval fish foraging and growth in a transparent, mechanistic, and generic model, and thus facilitates ecologically meaningful comparisons between different early life history strategies and different environments.

### Application

The present study quantifies how well Quirks depicts the foraging and growth of marine fish larvae. Traits were chosen to characterize larvae 2 to 4 mm beyond the length of yolk depletion, which we refer to as “young larvae”. This size range was chosen for various reasons. First, the growth dynamics of larvae with yolk reserves are dominated by the quantity of yolk and the effect of temperature on the conversion of yolk to somatic tissue, as opposed to the success of foraging on prey organisms [12], [13]. Second, larvae are most susceptible to starvation and have the most specific prey requirements (high concentrations of small prey) immediately after yolk depletion, due to the combination of small size and obligate exogenous feeding [14]–[16]. Third, data on first-feeding larvae are sparse and difficult to interpret, because rapid behavioral and physiological changes occur during this stage of development. Our compromise was not to model first-feeding larvae, but young larvae that are still small, yet already accomplished predators, for which more and better empirical data were available.

**Figure 1:**
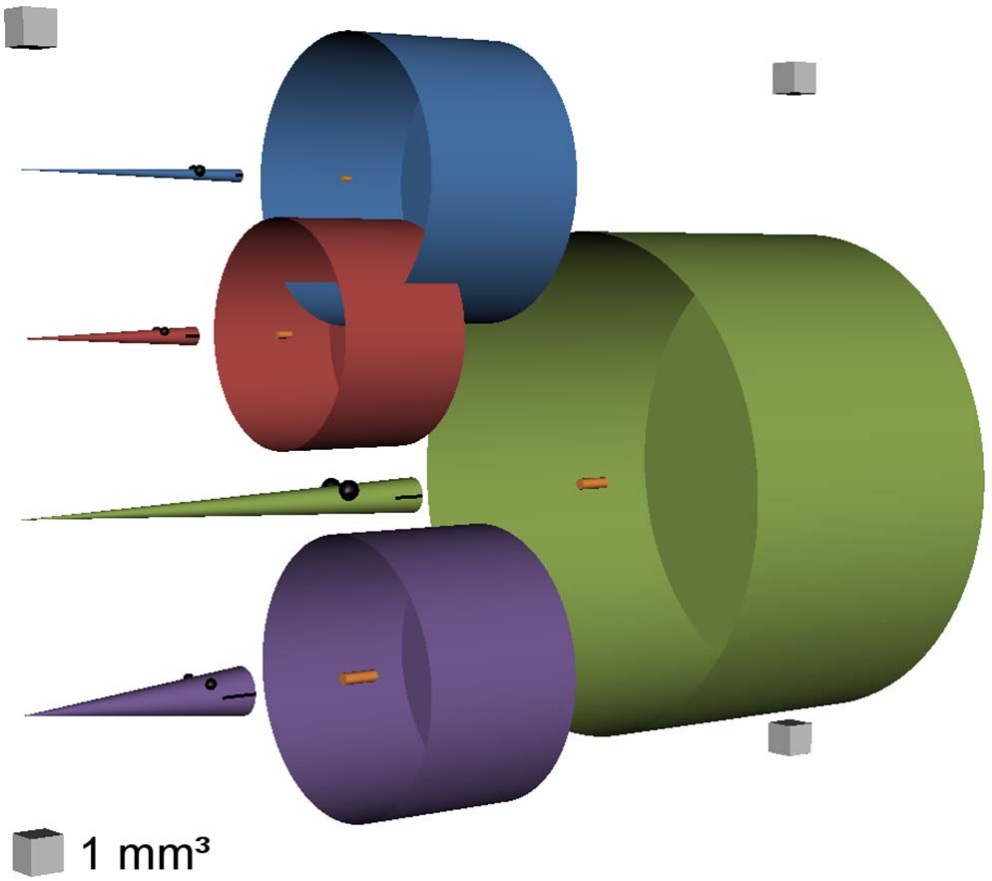
Morphological and behavioral traits of modeled fish larvae. This comic illustrates the four types of modeled fish larvae (cones), their effective visual fields (open cylinders), and their maximum ingestible prey (orange). Standard length, maximum prey length (additionally represented by mouth length), visual field radius, and encounter distance are rendered to scale (1 mm^3^ gray cubes). Visual field length and volume represent 1 s of swimming (mm s^−1^) and foraging (mm^3^), respectively. Fish and prey volume are scaled such that 1 mm^3^ = 100 µg dry mass (tissue of 91% water content and 1 g ml^−1^ density). Blue: 7-mm sprat (*Sprattus sprattus*), red: 5-mm anchovy (*Engraulis encrasicolus*), green: 13-mm herring (*Clupea harengus*), purple: 7-mm cod (*Gadus morhua*).

We parameterized Quirks based on the characteristics of young larvae of four marine fish species inhabiting the North Sea: “anchovy” (European anchovy, *Engraulis encrasicolus*), “cod” (Atlantic cod, *Gadus morhua*), “herring” (Atlantic herring, *Clupea harengus*), and “sprat” (European sprat, *Sprattus sprattus*). These species provide several contrasts that are important to the development of a generic model. First, they differ in developmental morphology. For example, the standard length by which 95% of larvae deplete their yolk reserves varies three-fold, and the dry mass of young larvae of the same length varies 15-fold (Figure 1) [17]–[19]. Second, the four species differ in their biogeographic distribution. The North Sea is near the lower latitudinal limit for cod and herring in the northeast Atlantic (sub-Arctic to temperate species favoring relatively cool waters), whereas it is the higher latitudinal limit for sprat and anchovy (temperate to sub-tropical species favoring relatively warm waters). Quirks is not specific to the North Sea or any other ecosystem, and the chosen species allowed us to use field data from many other locations (e.g., the northwest Atlantic, northeast Arctic, Baltic Sea, and Mediterranean Sea) to validate the model. Third, each species has a different spawning period in the North Sea: cod (winter to spring), sprat (spring to summer), anchovy (summer), and herring (autumn to spring) [20]–[23]. Consequently, young larvae experience environmental conditions that vary in temperature from <5 to >15°C and in photoperiod from <12 to >16 h of daylight in the North Sea (and to even greater extremes in other locations) [24], [25]. Finally, the four species span three different families (*Clupeidae*, *Engraulidae*, and *Gadidae*), which display different behavioral and physiological characteristics shortly after first-feeding.

## Methods

Quirks was developed from larval fish IBMs for herring [23] as well as cod and sprat [26] that, in turn, were based on other previous models such as the generalized IBM by Letcher and colleagues [9] and the seminal model of cruise predation by Gerritsen and Strickler [27]. Therefore, many processes were represented by equations familiar to (larval fish) ecologists and modelers. However, model development also involved a thorough review of empirical studies and measurements of larval fish behavior and physiology. In synthesis of this literature, we developed several new, simplified, or generalized equations. To facilitate comparative modeling, different types of larvae were fully defined by a list of traits (constants, Table 1), while all biophysical processes were modeled using one unified set of rules (equations, Table 2). Finding appropriate values for many traits required the re-interpretation of published laboratory and field data [9]. An unusual aspect of Quirks was that, under highly favorable conditions (warm temperature, long photoperiod, high prey concentration), larvae quickly grew beyond the intended 2-mm length range of the model parameterization. Slightly smaller or larger larvae must have quite similar traits, but we did not attempt to quantify this in the present study. Instead, we ensured that larvae did not outgrow their size-specific traits, by limiting each simulation to a period of 24 h. These short simulations provided snapshots of modeled ecology much like field sampling produces snapshots of ecology *in situ*, and were thus well suited for model validation.

**Table 1:** Biological traits of modeled anchovy (*Engraulis encrasicolus*), cod (*Gadus morhua*), herring (*Clupea harengus*), and sprat (*Sprattus sprattus*) larvae.

Symbol	Description	Unit	Anchovy	Cod	Herring	Sprat	References
*x*_body_	Body shape	µg mm^−3^	0.226	1.04	0.168	0.0684	[51] b, [19] b, [23] b, [19] b
*x*_ing_	Ingestible prey length	(*L*)	8.05%	15.70%	7.84%	3.84%	[86] b, [87] b, [23] b, [88], [89] b
*x*_len_	Initial standard length^a^	mm	5.5	7	13	7	[18], [90], [17], [88]
*x*_res_	Routine respiration at 10°C	(*M*) h^−1^	4.11%	2.03%	2.40%	2.40%	[32], [91] ^bc^, [92] b, [23] b, d
*x*_rQ10_	Respiratory Q10		1.39	2.38	1.71	1.71	[32], [91] ^bc^, [92] b, [23] b, d
*x*_tol_	Upper thermal tolerance	°C	≥25	15	20	16	[93], [94], [94], [95]
*y*_act_	Cost of foraging activity	(*R*)	200%	200%	200%	200%	[3] e
*y*_det_	Prey length detected to 50%	mm	0.07	0.07	0.07	0.07	[15] b
*y*_dig_	Digestion at 10°C	(*M*) h^−1^	2.50%	2.50%	2.50%	2.50%	[23], [32], [61], [92], [96]–[99] e
*y*_dist_	Encounter distance	(*L*)	50%	50%	50%	50%	[49], [50], [66], [68], [70] e
*y*_dQ10_	Digestive Q10		2.5	2.5	2.5	2.5	[32], [61], [100], [101] e
*y*_eff_	Metabolic efficiency		67.50%	67.50%	67.50%	67.50%	[28], [30], [32], [52], [102] e
*y*_hand_	Handling time	s	1.5	1.5	1.5	1.5	[49], [50], [63], [65], [66], [68], [70] e
*y*_swim_	Swimming speed	(*L*) s^−1^	75%	75%	75%	75%	[55], [62], [66]–[68], [70], [103] ^be^
*y*_turb_	Corrigible turbulent velocity	(*L*) s^−1^	100%	100%	100%	100%	[49] b
*y*_vis_	Effective visual cylinder radius	(*L*)	60%	60%	60%	60%	[15], [50], [55], [62]–[70] ^be^

*L*: standard length, *M*: dry mass, *R*: routine respiration.

a Standard length at yolk depletion +2 mm.

b Based on size-specific re-analysis of published data.

c *E. encrasicolus* parameter estimated from *E. mordax* or *A. mitchilli* data.

d *S. sprattus* parameter estimated by *C. harengus* parameter.

e Approximate median for multiple studies and species.

**Table 2:** Quirks model equations.

Number	Symbol	Description	Unit	Definition
(1)	*G*	Specific growth rate	d^−1^	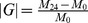
(2)	*L*_0_	Initial standard length	mm	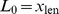
(3)	*M*_0_	Initial dry mass	µg	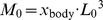
(4)	*L*	Standard length^a^	mm	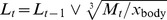
(5)	*M*	Dry mass	µg	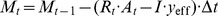
(6)	*R*	Routine respiration	µg h^−1^	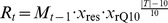
(7)	*A*	Activity		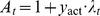
(8)	*I*	Ingestion^b^	µg h^−1^	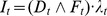
(9)	*D*	Digestive capacity	µg h^−1^	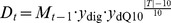
(10)	*c*	Prey concentration	mm^−3^	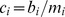
(11)	*b*	Prey biomass conc.	µg mm^−3^	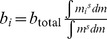
(12)	*m*	Prey item dry mass^c^	µg	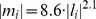
(13)	*F*	Foraging capacity^d^	µg s^−1^	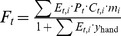
(14)	*E*	Encounters with prey	s^−1^	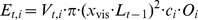
(15)	*V*	Combined velocity	mm s^−1^	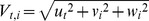
(16)	*u*	Predator velocity	mm s^−1^	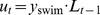
(17)	*v*	Prey velocity^e^	mm s^−1^	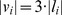
(18)	*w*	Turbulent velocity^f^	mm s^−1^	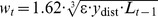
(19)	*O*	Observation success		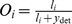
(20)	*P*	Pursuit success^a^		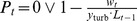
(21)	*C*	Capture success^a^		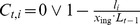

a “∨” denotes the “max” function operator (e.g., 1 ∨ 2 = 2).

b “∧” denotes the “min” function operator (e.g., 1 ∧ 2 = 1).

c Based on 0.04 to 2 mm protist plankton and copepods [39]–[42].

d Numerically maximized assuming optimal diet composition.

e Primarily based on zooplankton <1 mm [104], [105].

f 1.62≈55/18 of the universal Kolmogorov constant [44]–[47].

### Model Structure

#### Nomenclature

To ease the understanding of mathematical notation, we applied the following rules of nomenclature. Larval traits were denoted by *x* if treated as species-specific and by *y* if treated as generic, with subscripts abbreviating the role of the trait. Variables with the subscript *t* were re-calculated every time-step. Variables with the subscript *i* were calculated separately for each size bin of potential prey items.

#### Growth physiology (equations 1–9)

To calculate growth, the two state variables standard length *L* and dry mass *M* were initialized, then a series of equations was iteratively solved for 24 hourly time steps, one night followed by one day (Tables 2–3). Initial *L* was set to *x*_len_. Initial *M* and positive *L*-growth were determined according to the body shape trait *x*_body_, which functioned as an upper limit to *M*/*L* ^3^ and a reference point for larval condition (*M*/*L* ^3^ is often termed Fulton’s condition factor). A loss of *M* never resulted in a loss of *L*. All *L*-changes were thus modeled as isometric growth, an approximation simplifying direct comparisons of *x*_body_ among different larval types. Changes in *M* were calculated from the balance of metabolic gains from ingested prey (with metabolic efficiency *y*_eff_) and losses to active respiration. Respiration was modeled as a percentage of *M* lost per hour at 10°C (routine respiration *x*_res_), corrected for ambient temperature by a Q_10_ factor (*x*_rQ10_). We converted respiration from units of oxygen uptake to units of dry mass using a conversion factor of 0.85 µg µl^−1^, which is typical for young marine fish larvae [28]–[33]. Respiration was increased between sunrise and sunset to account for the cost of foraging activity (*y*_act_). The ingestion of prey was limited by the lesser of either digestive capacity or foraging capacity during the daylight fraction of the time step. Digestive capacity was represented as a percentage of *M* evacuated per hour at 10°C (*y*_dig_) corrected for ambient temperature by a Q10 factor (*y*_dQ10_). Larvae encountering temperatures in excess of their upper thermal tolerance (*x*_tol_) were excluded.

**Table 3:** Quirks environmental factors and internal variables.

Symbol	Description	Unit	Range
*b*_total_	Total prey dry biomass	µg mm^−3^	0 to ∞
*ε*	Turbulent kinetic energy dissipation rate	W kg^−1^	10^−7^ unless noted
*i*	Prey bin index		1, 2, …, 196
*l_i_*	Prey length	mm	0.045, 0.050, …, 1.95
*λ_t_*	Light (fraction of *t* with daylight)		0 to 1
*s*	Normalized size spectrum slope		−1.2 unless noted
*t*	Time step	h	1, 2, …, 24
*T*	Temperature	°C	0 to *x*_tol_

#### Prey fields (equations 10–12)

Potential prey were binned into groups of characteristic length *l_i_*, individual dry mass *m_i_*, biomass concentration *b_i_*, and numeric concentration *c_i_*. The relationship among these variables was idealized as a normalized size spectrum *sensu* Platt and Denman [34], by assuming a linear slope *s* between the logarithm of normalized *b_i_* (i.e., *b_i_* divided by the width of the *m_i_* bin) and the logarithm of *m_i_*. This simplification was consistent with empirical data from diverse aquatic systems [35]–[37] and a growing body of literature on size spectrum theory [34], [36], [38]. Prey fields were specified in terms of *l_i_* bins, *b_total_*, and *s*. For each bin, *c_i_* was equal to *b_i_* divided by *m_i_*. The distribution of *b_total_* among prey size bins (according to *s*) was solved analytically. The present study used prey from 0.04 to 2 mm length in 196 discrete 0.01-mm bins. A power function fit to this size range of copepods [39]–[41] and protist plankton [42] was used to calculate *m_i_* (n = 109, r^2^ = 0.95, two outliers removed, 45% carbon content assumed when necessary).

#### Foraging behavior (equations 13–21)

Quirks modeled foraging capacity based on a predation sequence of proximity, encounter, pursuit, attack, and ingestion, and assumed optimal diet selection (accounting for handling time *y*_hand_) [9], [43]. Proximity: The number of prey passing in proximity of larvae was calculated from prey concentration multiplied by the volume of a visually scanned cylinder. The radius of the cylinder represented larval visual ability (*y*_vis_). The length of the cylinder represented the combined velocities of larval swimming (*y*_swim_), prey swimming, and turbulence. Relative turbulent velocity between predator and prey (separated by encounter distance *y*_dist_) was calculated according to “Kolmogorov 1941 theory” [44], [45], assuming a universal Kolmogorov constant of 0.53 [46], [47]. While this approach is theoretically only valid for larger separation distances, it has been empirically validated at the scale of larval fish predation events [48], [49]. Encounter: In addition to proximity, encounters required prey detection (observation success), which was varied as a type 2 functional response with half-detected prey length *y*_det_, based on herring larvae reacting to prey of different sizes [15]. Pursuit: As observed in data of cod larvae feeding under turbulent conditions [49], relative pursuit success was linearly reduced from 100% at zero turbulence to 0% at a maximum corrigible turbulent velocity *y*_turb_. Capture: Based on herring larvae attacking prey of various sizes [15], relative capture success was linearly reduced from 100% for infinitesimally small prey length to 0% for the maximum ingestible prey length (*x*_ing_).

### Model Parameterization

#### Larval traits

Estimates for all traits were open to interpretation, due to the uncertainty, variability, and availability of empirical data. Some of the variability among published measurements reflected different environmental conditions. To realistically model maximum growth potential, measurements representing favorable conditions were used for traits related to morphology, such as larval shape (relating growth and condition) or ingestible prey size. To realistically model prey-limited growth and starvation, measurements characterizing low prey concentrations were used for foraging-related traits, such as swimming speed or metabolic efficiency (high efficiency for low rates of ingestion). In some cases, contradictory results were found in the literature, and averaged parameter estimates were used. Only six traits were deemed adequately studied and sufficiently variable among species to merit species-specific parameterization. Of these six, the traits *x*_res_ and *x*_rQ10_ were estimated for *E. encrasicolus* based on measurements in two closely related species: Northern anchovy (*Engraulis mordax*) and Bay anchovy (*Anchoa mitchilli*). For young sprat larvae *x*_res_ and *x*_rQ10_ were estimated by the herring parameters.

#### Generic digestion

Quirks represented the complex process of digestion in a greatly simplified, mechanistic form. Conceptually, *M*-gain from digestion depended on how much prey fit inside the larval gut at one time, how quickly the gut contents were digested and evacuated (temperature dependent), and an efficiency term accounting for losses to excretion as well as the costs of digestion and growth. A literature review of these factors (Table 1) yielded too little information for species-specific parameterization, so generic traits were used for digestion. The trait *y*_eff_ = 67.5% combined an assimilation efficiency of 75% and specific dynamic action of 10% (75% · 90% = 67.5%). The trait *y*_dig_ = 2.5% incorporated a gut capacity of 5% *M* divided by an evacuation time of 2 h. For every 10°C increase in temperature, the gut evacuation rate was multiplied by a Q10 factor of *y*_dQ10_ = 2.5.

#### Volumetric foraging rate

Parameterizing the rate at which larvae effectively scanned their environment for prey was of particular importance. Two main types of estimates were available from the literature: the multiplication of visual fields by swimming speeds and the division of predator reaction rates by prey concentrations. In studies using the multiplication approach, video recordings of larval reactions to prey were used to measure the size and shape of larval visual fields. Then, the continuous (cruise predator) or saltatory (pause-travel predator) movements between successive visual fields were used to calculate volumetric foraging rate. This method generally involves the unrealistic assumption that larvae actually detect 100% of the potential prey organisms passing through their visual fields [50], and is likely to overestimate foraging rate. In studies using the division approach, the rate at which larvae pursued, attacked, or ingested prey at very low concentrations was recorded. Then, volumetric foraging rate was estimated by dividing the mean reaction rate (e.g., the number of strike postures per minute) by the prey concentration. This approach is likely to underestimate foraging rate, if any prey are detected but not pursued, pursued but not attacked, or attacked but not ingested. Various other sources of bias likely affect both approaches (prey type, size, and concentration; experimental water volume, turbidity, and turbulence; larval size, concentration, condition, and satiation). In synthesis of available experimental data for species of anchovy (*A. mitchilli* and *E. mordax*), cod, and herring, we chose a generic effectively scanned cylinder radius of *y*_vis_ = 0.6 *L* s^−1^ and a generic effective swimming speed of *y*_swim_ = 0.75 *L* s^−1^. This resulted in foraging rates of 0.5 l h^−1^ for 5.5-mm anchovy, 1.0 l h^−1^ for both 7-mm cod and sprat, and 6.7 l h^−1^ for 13-mm herring larvae (not accounting for prey swimming, turbulence, or imperfect prey detection). For an illustration of the four larval types and their foraging rates, see Figure 1.

### Model Runs

#### Validation

We compiled a list of empirical studies estimating growth rates of young anchovy, cod, herring, and sprat larvae (Table 4), and used it to validate Quirks results. Data used for validation were independent from data used for parameterization except for one study yielding anchovy body shape [51] and one of five studies yielding generic metabolic efficiency [52]. Studies comparing different environmental conditions (including starvation experiments), reporting particularly high growth rates, or applying alternative methodologies were preferentially selected, and all those allowing for temperature, photoperiod, and prey concentration estimates were used. One sprat study by Dulčić [53] was included under the explicit assumption that growth had not been prey limited. The assumption was warranted, since Dulčić [53] replicated essentially identical and high growth measurements in two different years, despite a relatively cold temperature and a short photoperiod. The validation list included field and laboratory growth estimates based on larval length, mass, or protein content, otolith microstructure, and biochemical condition (RNA-DNA ratio). Quirks was set up to mimic the environmental conditions of each study and estimate growth. The slope *s* of the normalized size spectrum could not be calculated for most studies and was otherwise set to a default value of −1.2. This number has frequently been used as a reference point [36] ever since it was predicted from theoretical considerations of oligotrophic aquatic systems in equilibrium [34], [36]. For field studies, turbulent kinetic energy dissipation rate *ε* was set to 10^−7 ^W kg^−1^, approximating wind-driven turbulence at 30 m depth in storm conditions or near the surface in calm conditions [54], and sufficient to significantly influence larval fish foraging success [49], [55]. The default for laboratory studies was zero turbulence. Temperature was set to match experimental treatments (lab) or the surface layer (field). For several studies, photoperiod was estimated from date and latitude using the NOAA solar calculator, as implemented in the R package “maptools” [25]. For field studies, prey concentrations were matched to larval growth rates on a station-by-station basis when possible; otherwise, median prey concentrations were used.

**Table 4:** List of studies used to validate Quirks growth rate estimates.

Species	Location or Stock	Study	Growth estimate by	Sample size for validation	References
Anchovy	Adriatic Sea	Lab	Date, length	1	[106]
Anchovy	Catalan Sea, Gulf of Lions	Field	OIN, length	2 (regions)	[107]
Anchovy	N Catalan Sea	Field	OIN, length	1	[108]
Anchovy	Aegean Sea	Field	OIN, length, mass	4 (2 years×2 months)	[51]
Cod	Rhode Island	Lab	Date, mass	3 (temperatures)	[109]
Cod	W Norway, NE Arctic	Lab	Date, mass	6 (temperatures)	[57]
Cod	Georges Bank	Field	RNA:DNA	2 (years)	[92]
Cod	Newfoundland	Lab	Date, mass	3 (photoperiods)	[56]
Cod	Gulf of Maine	Lab	Date, protein content	6 (prey & temp. treatments)	[110]
Herring	Clyde, Downs, Limfjord	Lab	Date, mass	7 (5 prey conc., 3 stocks)	[52], [64]
Herring	W Norway	Lab	Date, mass	2 (photoperiods)	[111]
Herring	W Norway	Lab	Date, mass	2 (temperatures)	[112]
Herring	Baltic	Field	RNA:DNA	7 (2 sites, 5 weeks)	[73]
Sprat	German Bight	Lab	OIN, length	1	[113]
Sprat	E North Sea	Field	OIN, OS	4 (sites)	[114]
Sprat	N Adriatic Sea	Field	OIN, length	2 (years)	[53]

OIN: otolith increment number, OS: otolith size.

#### Sensitivity analysis

We measured the influence of endogenous larval traits and exogenous environmental factors on Quirks output in two series of individual parameter perturbations [5]. The first series quantified the sensitivity of modeled growth potential assuming *ad libitum* feeding. The second series examined parameter influence on the prey concentration required for *M*-growth of exactly 5% d^−1^. For each parameter and larval type, we determined the parameter range for which the output changed by less than ±10%. For example, model sensitivity to a hypothetical trait *x*_hyp_ could be such that any value between 80% and 200% of *x*_hyp_ results in similar growth potential (*G*±10%). Perturbations were performed with respect to species-specific reference points, representing the environmental conditions that young exogenously feeding larvae would typically experience in the central North Sea. Temperature and photoperiod were set to average conditions two weeks after the approximate midpoint of the adult spawning season (Table 5). Turbulence and prey fields were approximated by *ε* = 10^−7 ^W kg^−1^ and *s* = −1.2, as in the validation runs. Since prey concentration was fixed in the first sensitivity analysis (to ensure *ad libitum* feeding), and functioned as the output variable of the second analysis, the importance of prey concentration was separately quantified. This was done by determining the minimum level required for positive growth (starvation point), 5% growth, and maximum growth (satiation point).

**Table 5:** Comparison of anchovy (*Engraulis encrasicolus*), cod (*Gadus morhua*), autumn-spawned herring (*Clupea harengus*), and sprat (*Sprattus sprattus*) larvae in the North Sea.

Larval fish			Central North Sea Conditions			Prey requirement^a^ (mg m^−3^)			*G* (d^−1^)
Type	*L* (mm)	*M* (µg)	Date^b^	*T* (°C)^bc^	Photoperiod (h)^d^	Starvation	*G* = 5% d^−1^	Satiation	
Anchovy	5.5	38	August 1	15.0	15.9	7.4	8.8	12	16%
Cod	7	358	March 15	5.1	11.8	7.1	13	15	6.5%
Herring	13	369	October 1	14.7	11.6	2.7	3.8	6.3	17%
Sprat	7	23	May 15	8.9	16.0	1.4	2.0	2.8	13%

a Total dry mass of plankton in the length range from 0.04 to 2 mm.

b Estimated from adult spawning season [20]–[22], [115].

c Estimated from World Ocean Atlas 2009 [24].

d Calculated for a latitude of 54.5° N [25].

*L*: standard length, *M*: dry mass, *T*: temperature, *G*: specific growth rate.

#### Optimal foraging conditions

Here, we quantified the suitability of different prey sizes, prey-size distributions, and turbulence levels (increasing turbulence enhances encounters with prey, but decreases pursuit success) for young larvae. First, we examined encounters with each of the 196 modeled prey bins, in terms of the average ingested dry mass per encounter, standardized by handling time. This metric is used in foraging theory (and in Quirks) to determine the ranking of different prey types for inclusion in an optimal diet [9], [43]; from a predator’s perspective, it determines the optimal prey length. Second, we calculated a numerical solution for the value of *s* (normalized size spectrum slope) resulting in the lowest possible starvation (and satiation) point for each larval type. In the idealized model framework, *s* fully defines the size distribution of available plankton biomass, and thus also the optimal prey field. To further examine the effects of turbulence, we also calculated starvation and satiation points for *ε*-values of 10^−12^ to 10^−4 ^W kg^−1^.

## Results

### Validation

**Figure 2:**
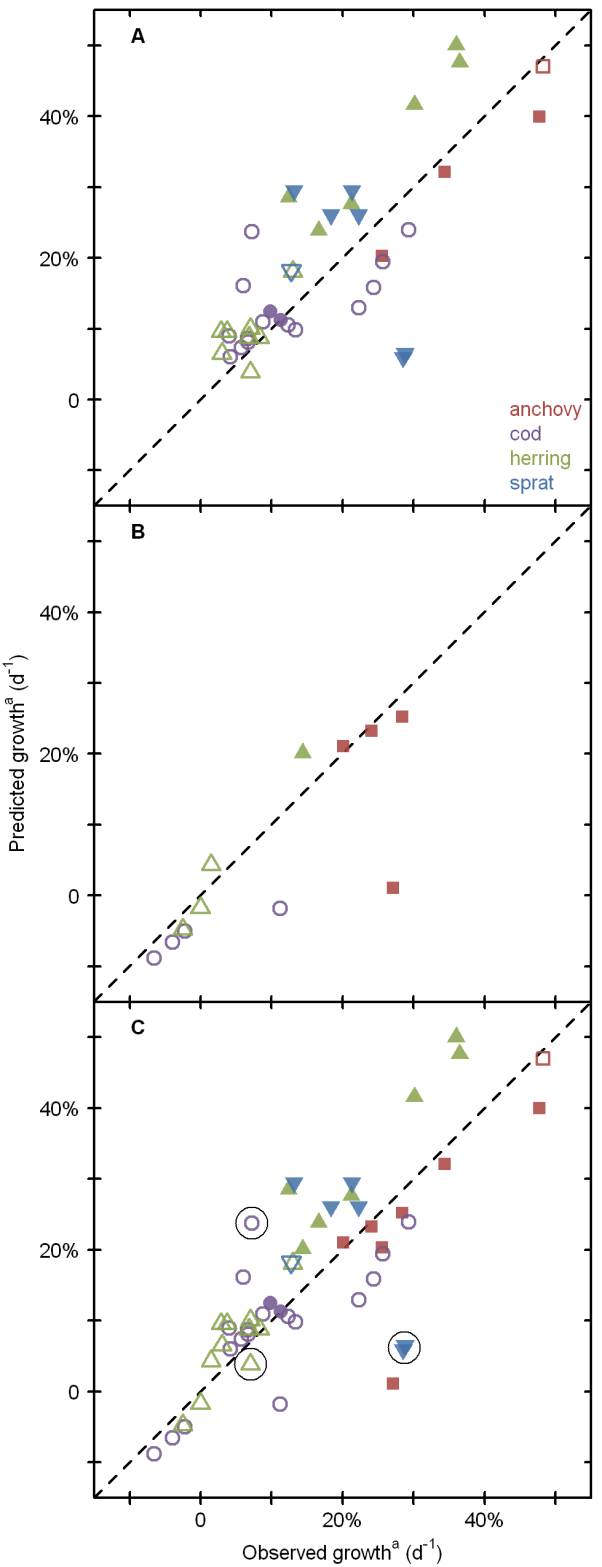
Validation of growth rates predicted by Quirks. For validation, 53 larval fish growth estimates from 17 publications were compared to model predictions for matching environmental conditions. Panel A shows the subset of data with prey concentrations that did not limit modeled growth. Panel B shows the subset in which modeled growth was prey limited. Panel C combines all data, and highlights four cases (circled) with extreme photoperiods (see text). Accurate predictions should fall near the dashed 1∶1 identity line (see table 6 for statistics). Empty symbols: laboratory studies, full symbols: field studies. Red squares: 5-mm anchovy (*Engraulis encrasicolus*), purple circles: 7-mm cod (*Gadus morhua*), green point-up triangles: 13-mm herring (*Clupea harengus*), blue point-down triangles: 7-mm sprat (*Sprattus sprattus*). ^a^Total dry mass of plankton in the length range from 0.04 to 2 mm.

A total of 53 growth estimates from 17 studies was used to validate Quirks output (Table 4). Prey concentration ranged from zero (cod, herring) to levels well above larval requirements (all species), temperature ranged from <3°C (cod) to >24°C (anchovy), and photoperiod ranged from <10 h daylight (cod, sprat) to continuous light (cod). We initially divided the data into two subsets: the “satiated” data with prey concentration in excess of the (modeled) satiation point and the “prey-limited” data with prey below the satiation point (Table 6, Figure 2). In the satiated subset (Figure 2A), there was a strong positive association between predicted and observed growth (Kendall rank correlation tau = 0.53, p<<0.001). Variability in the model estimate explained r^2^ = 58% of variability in the empirical estimate (linear regression method), and data scatter matched the 1∶1 identity line with R^2^ = 47% (sum of squares method). These statistics quantify model validity and skill in predicting growth across the four parameterizations (but not necessarily for any given one) under conditions of (modeled) *ad libitum* feeding. The prey-limited subset (Figure 2B), which did not include any sprat data, yielded equivalent statistics (R^2^ = 51%). Model performance was therefore not noticeably reduced under conditions of (modeled) prey limitation. Model performance was also equivalent for the complete dataset (R^2^ = 52%, Figure 2C). Prediction error (predicted minus observed growth) was independent of temperature (tau = 0.04, p = 0.66) but significantly associated with photoperiod (tau = 0.37, p<<0.001). For each hour of daylight above 13.4 h, Quirks tended to overestimate specific growth rate by 1.9% ±6.7% d^−1^ (median ± inter quartile range), and vice versa. We calculated model performance one more time, after excluding extreme photoperiods (n = 3 at ∼9 h daylight, n = 1 at 24 h daylight, circled in Figure 1C). The resulting “12 to 18 h daylight” subset matched the identity line to R^2^ = 65%, a marked increase over the other subsets (Table 6).

**Table 6:** Summary statistics quantifying model skill in matching published larval fish growth rates.

Subset	Data	Studies	Rank correlation		Linear fit	1∶1 line
	n	N	tau	P	r^2^	R^2^
Satiated	41	17	0.53	<<0.001	58%	47%
Prey-limited	12	5	0.79	<0.001	64%	51%
Combined	53	17	0.58	<<0.001	61%	52%
12 to 18 h daylight	49	16	0.68	<<0.001	72%	65%

### Sensitivity Analysis

Quirks output was highly sensitive to both endogenous traits and exogenous environmental factors (Tables 7–8). For growth potential to remain within ±10% of the reference values, the traits *y*_dig_ (digestion at 10°C) and *y*_eff_ (metabolic efficiency) could not be perturbed outside ∼95% to 105% of the original value for cod, herring, and sprat or outside ∼97%–103% for anchovy. The permissible range was up to ∼90%–110% for *x_res_* (routine respiration at 10°C) and ∼80%–120% for *y_act_* (cost of foraging activity). Growth potential changed by ±10% following photoperiod perturbations around ±6% in all species, but the sensitivity to temperature was quite species-specific. At one extreme, temperature influenced growth potential of young anchovy larvae more than any other parameter. At the other extreme, temperature was less influential than the five previously mentioned parameters for young cod larvae. Ranges for *y*_dQ10_ and *x*_rQ10_ (temperature dependency of digestion and respiration) were provided for the sake of completeness (Table 7), but primarily reflect whether water temperature was close to 10°C during the different spawning seasons. No other parameters influenced growth potential, unless larvae were rendered entirely unable to forage (e.g., due to zero vision or unmanageable turbulence). On the other hand, all 20 parameters influenced the prey requirement for 5% growth (Table 8). Here, the most critical parameters were effective visual cylinder radius *y*_vis_ (all species) and normalized size spectrum slope *s* (sprat). Perturbing these parameters outside a range of ∼95–105% resulted in a >10% change in prey requirement. The next most important parameters, in approximate order of influence, were *s* (anchovy), *y*_eff_ (all), *x*_body_ (all), *y*_swim_ (all), *x*_res_ (all), *x*_len_ (all), and photoperiod (all), with permissible ranges between ∼93%–110% and 83%–127% depending on the parameter and larval type (Table 8).

**Table 7:** Ranges (%) of individual parameter perturbations resulting in <10% change in modeled growth potential of North Sea anchovy (*Engraulis encrasicolus*), cod (*Gadus morhua*), autumn-spawned herring (*Clupea harengus*), and sprat (*Sprattus sprattus*) larvae.

Parameter	Anchovy		Cod		Herring		Sprat	
	from	to	from	to	from	to	from	to
Photoperiod	94.7	105.2	93.5	106.4	93.7	106.2	93.5	106.4
Temperature^a^	96.7	103.1	79.0	118.9	94.8	104.8	91.6	107.7
*x*_res_	94.8	105.3	90.2	109.8	90.2	109.9	91.3	108.8
*x*_rQ10_	89.8	110.9	82.7	123.2	80.3	122.3	44.7	238.5
*y*_act_	90.8	109.4	80.3	119.8	80.0	120.3	84.7	115.5
*y*_dig_	96.6	103.4	95.1	104.9	95.2	104.8	95.4	104.5
*y*_dQ10_	93.3	106.8	90.8	110.7	90.0	110.4	65.6	156.2
*y*_eff_	96.6	103.4	95.1	104.9	95.2	104.8	95.4	104.5

a Perturbations with respect to temperature in °C.

**Table 8:** Ranges (%) of individual parameter perturbations resulting in <10% change in modeled prey requirement for 5% d^−1^ growth of North Sea anchovy (*Engraulis encrasicolus*), cod (*Gadus morhua*), autumn-spawned herring (*Clupea harengus*), and sprat (*Sprattus sprattus*) larvae.

Parameter	Anchovy		Cod		Herring		Sprat	
	from	to	from	to	from	to	from	to
Photoperiod	83.5	124.7	88.4	115.3	87.3	117.3	85.4	120.5
Prey field (*s*)	93.4	110.0	83.7	164.7	84.3	155.7	95.1	106.5
Temperature^a^	74.4	122.8	57.8	135.3	83.2	114.6	69.2	126.9
Turbulence (*ε*)	7.3	350.5	4.3	397.3	0	626.0	3.1	430.6
*x*_body_	90.2	109.8	90.5	109.4	90.8	109.0	90.1	109.9
*x*_ing_	85.7	120.8	78.6	135.4	79.7	133.5	89.2	114.8
*x*_len_ b	100^b^	119.3	100^b^	126.9	100^b^	114.3	100^b^	113.5
*x*_res_	88.2	111.8	83.0	116.9	87.6	112.2	86.2	113.8
*x*_rQ10_	77.8	125.0	72.9	146.0	75.4	127.9	29.3	409.4
*x*_tol_ a	60	∞	33.9	∞	73.5	∞	55.9	∞
*y*_act_	79.6	120.4	65.8	133.8	75.1	124.5	76.0	124.0
*y*_det_	76.1	124.4	71.3	130.0	71.2	130.0	78.4	121.9
*y*_dig_ c	75.4	∞	88.4	∞	64.8	∞	71.7	∞
*y*_dist_	0	350.5	0	397.3	0	626.0	0	430.6
*y*_dQ10_ c	56.8	∞	0	128.4	39.7	∞	0	2350
*y*_eff_	91.1	110.9	91.4	110.5	91.7	110.1	91.0	111.0
*y*_hand_	0	596.6	0	263.2	0	189.5	0	1183
*y*_swim_	90.2	111.9	90.2	111.9	90.7	111.4	90.4	111.6
*y*_turb_	72.6	186.0	69.3	218.6	59.4	610.8	68.3	231.5
*y*_vis_	95.3	105.4	95.3	105.4	95.3	105.4	95.3	105.4

a Perturbations with respect to temperature in °C.

b Initial lengths <100% fell outside the parameterized length range and were omitted.

c Inside this range prey requirement was independent of digestion, outside this range growth was <5%.

At the species-specific reference conditions, the starvation point prey requirement ranged from 1.4 (sprat) to 2.7 (herring) to 7.1 (cod) to 7.4 (anchovy) mg m^−3^ (Table 5). The satiation point was between 1.6 (anchovy) and 2.3 (herring) times as high as the starvation point, while intermediate prey concentrations resulted in positive, prey-limited growth.

### Optimal Foraging Conditions

**Figure 3:**
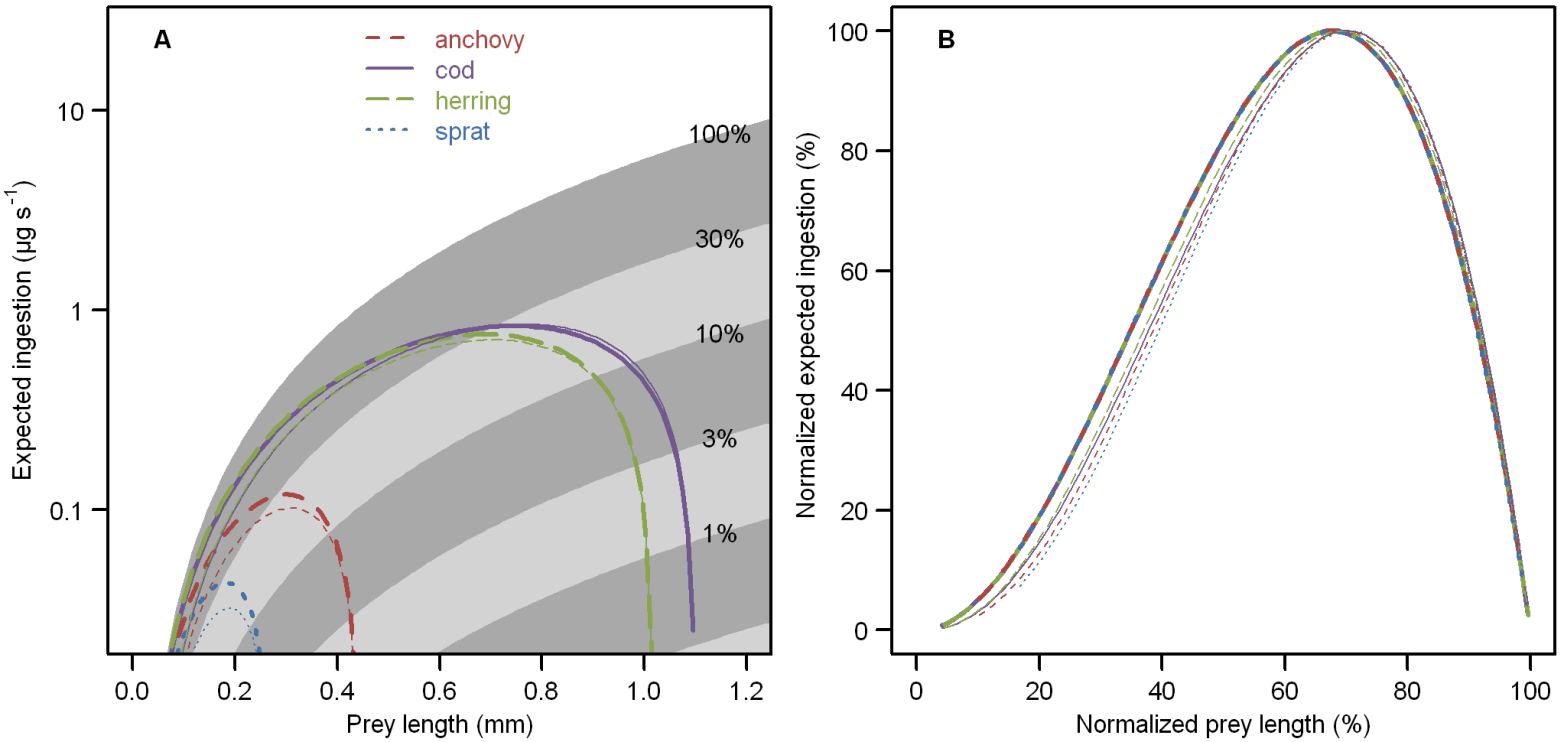
Suitability of different prey sizes for young anchovy, cod, herring, and sprat larvae. Encounters between young fish larvae and 196 size classes of plankton from 0.04 to 2(thick line) depended on pursuit success, capture success, prey dry mass, and handling time. The expected benefit per nearby prey organism (thin line) additionally “corrected” for imperfect observation success and the combined velocity of predator swimming, prey swimming, and turbulent velocity. Panel A shows ingestion and prey length in absolute units, highlighting dramatic differences among the four larval types. Panel B shows normalized values, revealing that the optimal prey length was ∼67% of the maximum ingestible length in all cases. Shading in A indicates the probability of successful pursuit and capture, as labeled. Medium-dashed red: 5-mm anchovy (*Engraulis encrasicolus*), solid purple: 7-mm cod (*Gadus morhua*), long-dashed green: 13-mm herring (*Clupea harengus*), short-dashed blue: 7-mm sprat (*Sprattus sprattus*). Turbulent kinetic energy dissipation rate was set to 10^−7 ^W kg^−1^.

**Figure 4:**
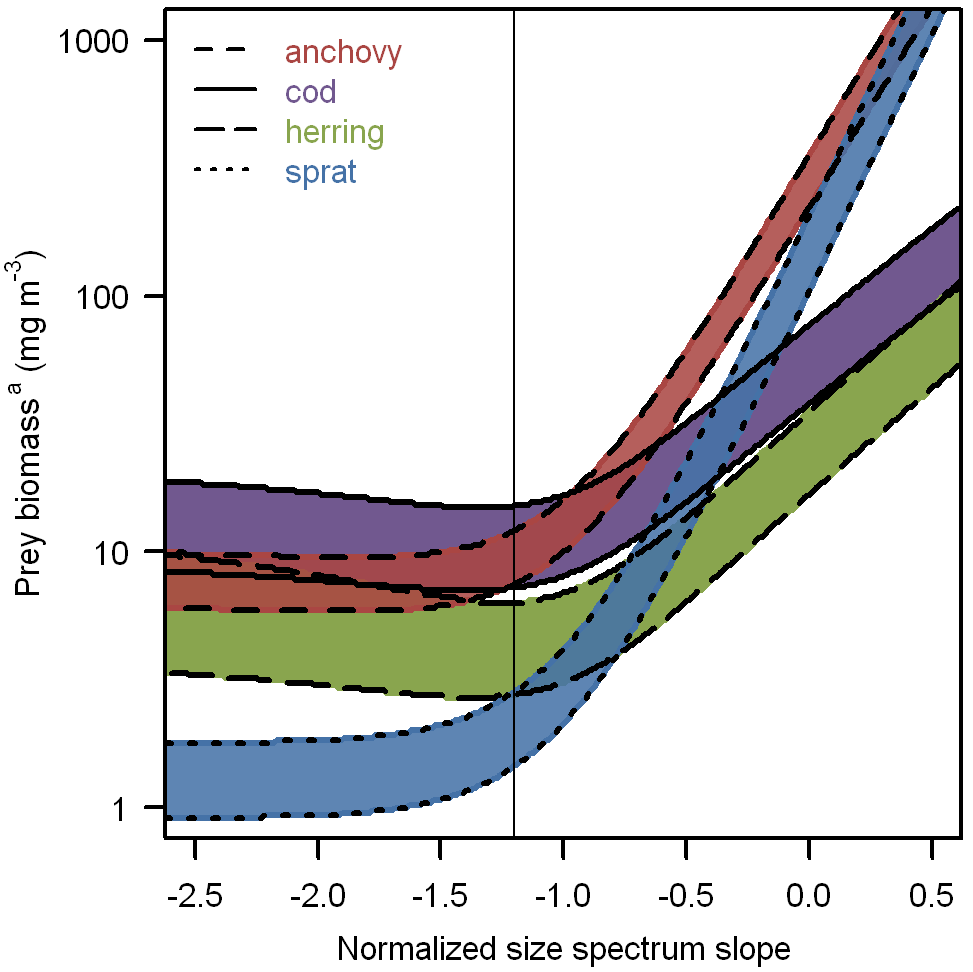
Starvation and satiation points of young fish larvae foraging in different prey fields. The minimum prey biomass^a^ that fish larvae required for positive growth and maximum growth was modeled for prey fields of varying normalized size spectrum slope *s*. At prey concentrations below the starvation point (lower line) larvae lost mass. Above the satiation point (upper line), larvae achieved their maximum growth potential. Intermediate growth rates occurred in the colored area between the starvation and the satiation points. The thin vertical line indicates the reference point of *s* = −1.2. Medium-dashed red: 5-mm anchovy (*Engraulis encrasicolus*), solid purple: 7-mm cod (*Gadus morhua*), long-dashed green: 13-mm herring (*Clupea harengus*), short-dashed blue: 7-mm sprat (*Sprattus sprattus*). ^a^Total dry mass of plankton in the length range from 0.04 to 2 mm.

**Figure 5:**
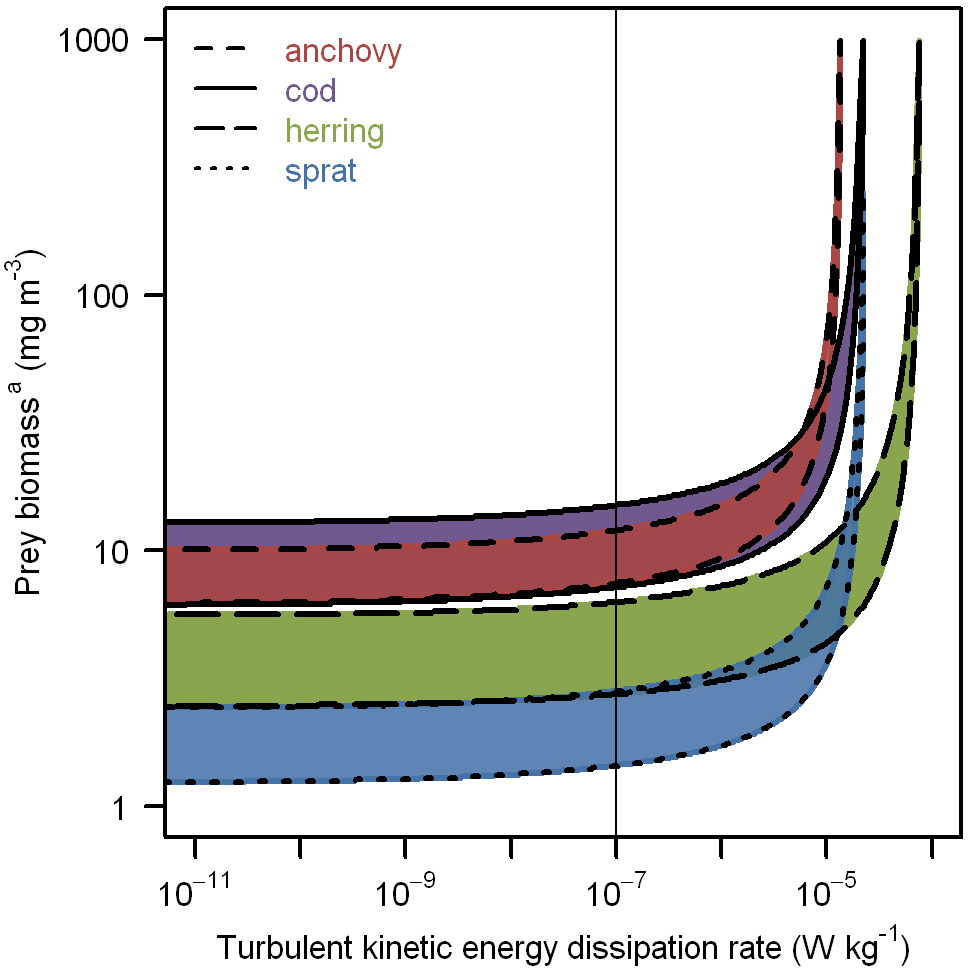
Starvation and satiation points of young fish larvae foraging at different levels of turbulence. The prey biomass^a^ that fish larvae experiencing various levels of turbulence required for growth was modeled. Maximum, negative, and intermediate positive growth rates occurred at prey concentrations above the satiation point (upper line), below the starvation point (lower line), and between the starvation and satiation points (colored area), respectively. The thin vertical line indicates the turbulence reference point used throughout much of the study. Medium-dashed red: 5-mm anchovy (*Engraulis encrasicolus*), solid purple: 7-mm cod (*Gadus morhua*), long-dashed green: 13-mm herring (*Clupea harengus*), short-dashed blue: 7-mm sprat (*Sprattus sprattus*). ^a^Total dry mass of plankton in the length range from 0.04 to 2 mm.

Optimal prey length primarily depended on maximum ingestible prey length, and was therefore smallest in sprat (0.18 mm), intermediate in anchovy (0.30 mm), and larger in herring (0.69 mm) and cod (0.74 mm) (Table 9, Figure 3). These values corresponded to 67%±0.4% (mean ± standard deviation) of the maximum ingestible prey length. The optimal normalized size spectrum slope *s* was –2.5 for sprat, –1.9 for anchovy, and –1.4 for herring and cod. While larvae could reach their full growth potential at any value of *s*, optimal size spectra resulted in reduced starvation and satiation points (Figure 4). Although turbulence had a positive influence on prey encounters and a negative influence on prey pursuit success, the overall effect was consistently negative. In all four larval types, starvation and satiation points increased monotonically with turbulent kinetic energy dissipation rate (Figure 5). Over 90% of the change occurred between 10^−8^ and 10^−4 ^W kg^−1^.

**Table 9:** Optimal foraging conditions for the indicated sizes of anchovy (*Engraulis encrasicolus*), cod (*Gadus morhua*), herring (*Clupea harengus*), and sprat (*Sprattus sprattus*) larvae.

	*L* (mm)	Maximum *l* (mm)	Optimal *l* (mm)	Optimal *s*	Optimal *ε* (W kg^−1^)
Anchovy	5.5	0.44	0.30	−1.9	0
Cod	7	1.10	0.74	−1.4	0
Herring	13	1.02	0.69	−1.4	0
Sprat	7	0.27	0.18	−2.5	0

*L*: standard length, *l*: prey length, *s*: normalized size spectrum slope, *ε*: Turbulent kinetic energy dissipation rate.

## Discussion

A solid understanding of the processes governing larval fish growth is of great scientific value. Even subtle differences in growth during fish early life history can result in dramatically different cumulative mortality and, ultimately, recruitment to adult populations [2], [3]. Evidence for prey-limited growth in nature is scarce, since it requires sampling at the appropriate spatial and temporal scales and across wide ranges in prey conditions. This is an additional reason why mechanistic models of larval fish foraging are in high demand [5]. Some field studies have successfully linked sub-optimal larval growth to low prey concentrations. For example, Buckley and Durbin [16] showed that the growth rates of <12-mm Atlantic cod and <7-mm haddock (*Melanogrammus aegelfinus*) on Georges Bank (northwest Atlantic) were strongly reduced in areas of low *in situ* prey concentration. Larger individuals captured at the same stations were not significantly affected. This is consistent with Quirks results: in all species, smaller larvae required higher prey concentrations than larger individuals, primarily because they scanned less water, and secondarily, because their capture success was lower. Buckley and Durbin’s [16] study illustrates that obtaining a large body size quickly, by rapid growth through the early larval stage, confers a clear survival advantage. Further, the authors concluded that rapid larval growth may be necessary (although not sufficient) for a strong year class [16]. In combination with the appropriate fisheries data, Quirks could be applied to study not only foraging and growth, but also the likelihood of strong year classes. While not addressed here, larval transport is another factor greatly influencing year-class strength, and there is a trend towards using coupled bioenergetic-transport models of fish larvae [4], [5]. Quirks can certainly be adapted for that approach, by defining traits that vary with growth and development.

### Validation

#### Model skill

In the validation runs, there was a good match between growth predicted by Quirks and growth observed empirically (R^2^ = 52%, Table 6, Figure 2). Further, the distribution of slopes between any two data points was centered near one (median = 0.72, interquartile range = 0.98), and the corresponding distribution of intercepts was centered at approximately zero (median = 0.04, interquartile range = 0.19). In other words, Quirks had surprisingly low bias (a slope of one and intercept of zero would indicate no systematic bias whatsoever). We did observe a statistically significant photoperiod effect, thus predicting growth at extreme photoperiods may require extra care. For example, Quirks predicted much too low growth potential (∼6% vs. ∼29% d^−1^) for sprat larvae in the northern Adriatic Sea in January (∼11°C, ∼9 h daylight) [53]. At the other extreme, Quirks predicted a growth rate of 24% vs. the observed 7% d^−1^ for cod larvae reared in continuous daylight at 8°C [56]. Here, modeled growth potential may not have been grossly inaccurate, because another study of cod larvae at the same 8°C temperature (and ∼15 h daylight) measured a growth rate of 22% d^−1^ [57]. In any case, model skill was noticeably higher among the “12 to 18 h daylight” subset of data (R^2^ = 65%, Table 6), which excluded the above outliers and one other (unproblematic) data point. Note that we did not “tune” the model equations or larval traits to optimally fit the validation dataset, and that we used almost entirely independent data for parameterization and validation (there was overlap with respect to one trait in two studies).

#### Growth

The list of validation studies encompassed a wide range of different approaches (Table 4), each subject to potential bias and random sampling error. For example, many lab studies are biased towards low growth, because larvae rarely thrive as well in artificial environments as they might under ideal natural conditions. Field studies, on the other hand, are likely to overestimate growth. For example, among simultaneously occurring and equally sized larvae, faster-growing individuals have, so far, suffered a shorter period of exposure to predation [2], [58], and may additionally have experienced lower instantaneous predation rates [59], [60]. Random sampling of the survivors is thus not sufficient for an unbiased characterization of the original population. Attempting to correct for these and other sources of uncertainty was beyond the scope of the present study, as was the distinction among individual growth rates (e.g., RNA-DNA ratios), “vertical” or “horizontal” population growth rates (i.e., based on patterns among simultaneously collected larvae or time-series of samples, respectively). Conceptually, it should be clear that the presented parameterizations represent average individuals, and that Quirks was used to model individual growth rates.

#### Prey concentration

Matching (i.e., simulating) empirical prey concentrations for the purpose of validation was also problematic. Given the complexity of plankton dynamics in the field, and the difficulty of maintaining nominal food levels in small-scale experiments, constant “matched” prey concentrations can only have roughly approximated the actual conditions experienced by larvae. For this reason we were surprised that Quirks performed equally well (in terms of R^2^) for the satiated and prey-limited subsets of validation data. The fact that 10 of 12 data points fell very near the 1∶1 line (Figure 1B) is encouraging, but should not be over-interpreted. Quirks underestimated growth in 9 of 12 cases (including 5 of 5 with empirically observed negative growth), which may indicate that our parameterization slightly overestimated larval respiration or slightly underestimated foraging capacity. In summary of model validation, a long list of uncertainties would have prevented even a perfect model from reproducing 100% of variability in the validation dataset. The observed R^2^ of up to 65% (Table 6) is therefore a conservative (low) estimate of Quirks’ model skill.

### Sensitivity Analysis

The sensitivity of model predictions to individual parameter perturbations is useful for several purposes. First, regarding Quirks in isolation, model sensitivity simply quantifies the influence of each trait and environmental factor on model output. This information helps potential users evaluate whether Quirks is a suitable tool for their questions and objectives. Second, with respect to larval traits estimated from the literature, model sensitivity quantifies the propagation of parameter uncertainty to model output. Any uncertain yet influential traits point to knowledge gaps particularly deserving of future research [5]. Third, sensitivity analysis is itself a form of model application that is well suited for the theoretical study of larval fish ecology [9]. Individual parameter perturbations essentially transform dissimilar processes into comparable units and provide a basis for conceptually difficult comparisons (e.g., between photoperiod and body shape). Finally, to the extent that Quirks is an accurate synthesis of larval fish ecology, model sensitivity also quantifies natural selection acting on potential larval fish phenotypes *in situ*. This information may shed light on the evolutionary ecology of fish early life history and help predict climate change effects. Note that systematically perturbing multiple parameters and interpreting the complex patterns of interaction effects can yield additional information about model sensitivity, including a more robust ranking of the most important factors.

#### Maximum growth

As expected, growth potential and prey requirement for 5% growth were sensitive to different parameters, and model sensitivity/parameter influence varied by larval type. Of the factors influencing growth potential, only respiration (traits *x*_res_ and *x*_rQ10_) and the environment (temperature and photoperiod shortly after peak spawning, Table 5) differed among species. All other traits were either generic or rendered inconsequential by the arbitrarily high food concentration. In all larvae, growth potential was more sensitive to *y*_dig_ and *y*_eff_ (given *ad libitum* feeding, both are simply coefficients of digestion), than to *x*_res_ and *y*_act_ (coefficients of total and active respiration, respectively). Young anchovy larvae were most sensitive to all of the above traits, due to their high mass-specific respiration. For example, anchovy required a mere 3.4% increase in *y*_dig_ to boost growth potential by 10% while the other species required 4.5% to 4.9%. Young cod and herring larvae were similar in their sensitivities, even though herring had ∼18% higher *x*_res_ and were exposed to ∼10°C warmer water. On the other hand, young sprat larvae were 5%–31% more sensitive than herring, despite effectively identical traits, because they experienced much longer photoperiods (>16 versus <12 h daylight). Relative temperature changes were most important for anchovy (at 15.0°C), followed by herring (at 14.7°C), sprat (at 8.9°C) and cod (at 5.1°C). In contrast, the parameter influence of photoperiod was independent of the absolute photoperiod reference values. These initially counterintuitive results highlight the importance of species-specific reference points (i.e., realistic environmental conditions).

#### Prey requirement

Unlike growth potential, prey requirement (for 5% growth) was sensitive to all endogenous traits and exogenous environmental factors. The influence of several important traits was similar among species. Prey requirement was approximately proportional to *x*_body_, *y*_eff_, and *y*_swim_, and approximately inversely proportional to *y*_vis_^2^, making *y*_vis_ the most influential trait in all cases. Perturbations of several other parameters affected prey requirement very differently. Notably, young sprat and anchovy were much more sensitive to *s* than cod and herring larvae. The same pattern was also apparent for the less influential traits *x*_ing_ and *y*_det_. To generalize this relationship, parameters differentially affecting access to small prey sizes are expected to be particularly influential in species with a developmental morphology similar to sprat and anchovy, i.e. small size at yolk depletion and small mouth gape. The influence of *x*_res_ and *y*_act_ was greatest in young anchovy and least in young cod larvae, primarily due to their vastly different ecophysiology. To generalize, larvae with low temperature-specific respiration rates in combination with cold environments (e.g., cod) are expected to be less sensitive to perturbations in *x*_res_ and *y*_act_ than larvae displaying the opposite pattern (e.g., anchovy). The influence of *x*_len_ perturbations was related to body shape, such that the thinnest larvae (sprat) were most sensitive, the somewhat less thin herring and anchovy were only slightly less sensitive, but the dramatically thicker cod were only half as sensitive. Neither *y*_hand_, nor the parameters related to turbulence (ε, *y*_dist_ and *y*_turb_) had a strong influence on prey requirement (see detailed discussion below). Since larvae only used from 64.8% (herring) to 88.4% (cod) of their full digestive capacity to achieve 5% growth, and inhabited waters well below their upper thermal tolerance, small changes in *y*_dig_ or *x*_tol_ were entirely without consequence.

#### Uncertainty

Uncertainty in many traits clearly exceeded the range affecting 10% changes in growth potential or prey requirement. However, our literature review uncovered too little data for a meaningful trait-by-trait assessment of uncertainty. Instead, we will highlight just a few traits and processes of particular interest. First, uncertainty in gut evacuation rate (used to parameterize *y*_dig_) and assimilation efficiency (the main component of *y*_eff_) primarily limited the reliability of growth potential estimates, thus better measurements of digestion would be invaluable. The importance of these factors for mechanistic larval fish IBMs is well known and has recently been reviewed in detail [5], [61]. Second, uncertainty in volumetric foraging rate (used to parameterize *y*_vis_) overshadowed all other factors influencing prey requirements, indicating a need for further experimental research in this area. For example, Rosenthal and Hempel [62] determined that 11- to 13-mm herring could search 3.1 to 5.2 l h^−1^ based on estimated swimming speeds and visual fields. For the same 11 to 13 mm length range, Munk and Kiørboe calculated 3.2 l h^−1^ from feeding strike rates and 9.2 l h^−1^ from attack posture rates [63], while additional feeding strike data by the same authors for ∼13 mm herring yielded rates from 1.6 to 3.1 l h^−1^ [15], [64]. Reanalysis of four studies of 6- to 8-mm cod resulted in estimates of 0.4 to 5.2 l h^−1^ [55], [65]–[67], while three estimates for 5.5-mm anchovy (based on *A. mitchilli* and *E. mordax*) ranged from 0.17 to 0.55 l h^−1^ [68]–[70]. Prey used in the above studies varied from 0.04-mm protozoans to 0.8-mm copepods, but 0.12- to 0.23-mm copepod nauplii were most common. Our results for 13-mm herring, 7-mm cod (and sprat), and 5.5-mm anchovy feeding on 0.2-mm prey were 5.0, 0.78, and 0.38 l h^−1^, respectively, and took into account prey swimming and detection of prey. Treating prey detection as a type 2 functional response to prey size (equation 19) was consistent with the above studies, in that it helped explain some of the variability among literature estimates. However, our estimate of *y*_det_ = 0.07 mm was based on a single study of 13- to 18-mm herring reacting to 0.1- to 0.8-mm copepods [15], thus the value is quite uncertain. Third, handling time estimates for anchovy and sprat provided a nice example of a highly uncertain parameter that was, nevertheless, fairly inconsequential for Quirks output. Mean handling time in seven studies of cod, herring, and closely-related species of anchovy (*E. mordax*, and *A. mitchilli*) varied from ∼0.5 to 3.5 s. It seems likely that the generic value of 1.5 s was perfectly adequate for anchovy and sprat, since any value below 8.9 s (anchovy) or 17.7 s (sprat) would have yielded very similar model output.

#### Natural selection

Our results led to several hypotheses regarding the evolutionary ecology of marine fish larvae. Due to the strong links between larval growth and survival [2], [3], [14], [58]–[60], phenotypes resulting in better growth are almost certainly favored by natural selection, yet the traits most likely to affect growth (assuming phenotypic variation among individuals) differ, depending on food limitation. When food is abundant, selection is likely to favor phenotypes with a fast metabolism, since the benefits of increased digestive capacity exceed the costs of increased respiration. There should also be selection for gains in digestive capacity or efficiency even at the expense of losses in foraging capacity or efficiency. In contrast, phenotypes with high foraging capacity and high gross growth efficiency (growth per ingestion) should be favored under food limitation. This reversal would result in selection towards slow metabolism and improved foraging (e.g., increased vision in all species, mouth gape in young anchovy and sprat) at the expense of digestive capacity or even metabolic efficiency.

Taking this line of reasoning a step further, we hypothesize that body plans prioritizing jaw and eye development over gut development may indicate species adapted to environments characterized by relatively low prey concentrations. In a previous study we estimated by principal component analysis that 16% of variability in the body shape of marine fish larvae involves a morphological gradient from a small head, eye, and jaw with a thick trunk to a large head, eye, and jaw with a thin trunk [3]. We may be able to use those data to test the above hypothesis. Given the strong influence of initial *L* on prey requirement, early life history strategies adapted to low-prey environments may also involve larger first-feeding larvae. This mechanism is independent from and synergistic with the finding that larger (longer) larvae can generally survive greater periods of starvation [14]. Note that the influence of *x*_body_ (body shape) perturbations is difficult to interpret in an evolutionary context, because changes in *L* affected both vision and mouth gape, while changes in *M* did not. Modeled larvae of enormous *L* and miniscule *M* would have a lower prey requirement than any realistic parameterization, following the computer science principle of “garbage in, garbage out.”

#### Absolute prey concentration

While it was easy to find species-specific temperature and photoperiod reference points for sensitivity analyses (Table 5), prey concentration would have been a more elusive variable. This is why we originally chose maximum growth potential as the first and prey requirement for fixed growth as the second sensitivity analysis output variable. Both metrics and analyses are, by design, independent of estimates of prey concentration. This does not change the fact that absolute prey concentrations below the satiation point had a strong negative influence on modeled growth (Table 5). In the range between starvation and satiation, the influence of prey concentration was roughly linear with 1 mg m^−3^ of plankton resulting in growth of 0.8% (cod), 3.5% (anchovy), 9.3% (sprat), and 4.7% (herring) d^−1^. Model sensitivity aside, it is interesting to compare absolute prey requirements of the four species. One might expect the requirements to be highest for larvae spawned in summer, when zooplankton biomass is highest in the North Sea [71]. Our model estimates do not agree with this expectation, since young cod larvae required a higher prey concentration in winter than sprat in spring, anchovy in summer or herring in autumn. This may simply reflect that our parameterizations were imperfect. Alternatively, the thermal tolerance of adults, eggs, or larvae may be more important in determining spawning seasonality than larval prey limitation.

### Optimal Foraging Conditions

#### Optimal prey size

Plankton at 67% of the maximum ingestible prey size constituted highly favorable prey for all four larval types. This pattern arose primarily from the trade-off between the decreasing probability of capture success and the increasing benefit of successful capture (Figure 3). Note that the chosen metric is independent of encounter rate, and therefore somewhat different from the expected benefit per nearby prey organism (e.g., in an aquarium). The latter can be slightly increased by prey movement or substantially reduced by low observation success (Figure 3). More variable optima have been reported in previous modeling studies, e.g.: 54% for 7-mm cod [26], 44% for 13-mm herring [23], and 85% for 7-mm sprat larvae [26]. Since those models applied different assumptions (equations) for each species, it is unclear whether this variability reflected differences among species or among models.

Empirical studies have rarely provided sufficient information to calculate the favorability of different prey sizes for young fish larvae. One exception was a series of laboratory experiments by Munk, where 13.5-mm herring larvae were fed low concentrations of copepods of various sizes [15]. In that study, the largest prey treatment (0.6 mm, ∼57% of maximum ingestible length) yielded the highest estimated ingestion per encounter (prey dry mass • capture success) as well as the highest ingestion rate (prey dry mass • capture success • attack rate). However, a much smaller prey treatment (0.2 mm, ∼19%) was considered optimal by the author, whose definition was based on the number of ingested copepods, as opposed to their mass. This logic permeates the literature, and is often quantified in terms of Chesson’s α, also known as Chesson’s (or Manley’s) measure of prey preference [72]. When applied to fish larvae, α should perhaps be called a measure of prey “sampling,” as the terms “preference” and “selection” are quite misleading. In fact, α neither reflects theoretical benefits (since prey mass is not considered), nor empirical behavior (since prey detection and capture success are assumed to be constant). Instead, it indicates sampling bias for different prey types, i.e. the relative probability of ingestion corrected by ambient numeric concentration (which is different from encounter rate). For comparison with Munk’s data, we calculated Chesson’s α for 13.5-mm herring larvae as simulated by Quirks: the prey length with maximal α was equivalent (0.21 mm, ∼21%). Note that the nutritional quality (e.g., fatty acid composition) of prey organisms also influences their benefit to larval fish predators [73]. This further level of detail was not considered in the present study, but could be modeled by “correcting” prey mass by a food quality coefficient in the determination of foraging capacity (equation 13).

#### Diet composition

Quirks assumes optimal foraging behavior, meaning that larvae only pursue and attack prey organisms when this is more profitable than spending the same time searching for better prey instead [9], [43]. Interestingly, optimal foraging was neither necessary for the model runs performed in this study, nor did it influence the results. In all cases, larvae achieved *ad libitum* feeding at prey concentrations far below the level at which they began actively excluding any ingestible prey from their diet. In other words, hungry larvae pursued everything from the smallest modeled prey class to the largest one they might possibly capture, while satiated larvae sometimes engaged in more complex selective feeding, but gained no benefit from doing so (because ingestion was limited by digestion). At later stages of development this would not hold true, and the explicit assumption of optimal foraging could become quite important.

#### Prey fields

Quirks’ representation of plankton size structure as normalized size spectra with independent biomass and slope *s*, presented an opportunity to determine optimal prey fields for each larval type (Figure 4). In all four cases, optimal prey fields were so “steep” that they consisted to >95% biomass of plankton smaller than the optimal prey size. This demonstrates that the integrated benefit of all prey sizes can be quite independent from the concentration of optimal prey. The optimal values of *s* for anchovy (−1.9), cod (−1.4), and herring (−1.4) fell within the range from 0 to −2, which is regularly observed in the nearby Bay of Biscay [37], while the optimum for sprat (−2.5) may be unrealistically steep [36]. In any case, there was relatively little change in prey requirements among such steep prey size spectra, while “flat” prey fields became increasingly unfavorable for young larvae (Figure 4).

#### Turbulence

Since the recognition that turbulence increases the rate of encounters between fish larvae and their prey [55], [74] but decreases subsequent pursuit success [49], [75], it has become common to include turbulence effects in individual-based models of larval fish foraging [5], [76], [77]. The relationship between foraging and turbulence is often assumed to be dome-shaped, with an optimal intermediate turbulence level that enhances encounter rates more than it reduces pursuit success. We can reproduce this shape in Quirks, but only for larvae swimming much more slowly than those discussed here. At the parameterized effective speeds of >4 mm s^−1^ the net influence of turbulence was always negative (Figure 5). In the laboratory, optimal intermediate turbulence has also been demonstrated only for small (poorly developed, slow) larvae [78]–[80]. In one study, small (<5 mm *L*, 7–9 d post hatch) Japanese flounder (*Paralichthys olivaceus*) larvae benefited significantly from turbulent kinetic energy dissipation rates between 10^−7^ and 10^−5 ^W kg^−1^, while slightly larger individuals (<7 mm, 12–14 d) did not [80]. A pattern of diminishing benefits of turbulence with increasing size (development, speed) also emerges from most models, with the pattern of decline depending on assumptions regarding visual field geometry, foraging behavior, pursuit success, and relative turbulent velocity *w* between predator and prey [67], [81]–[83]. As discussed, Quirks assumes a continuously but imperfectly scanned visual cylinder, and empirically validated Kolmogorov 1941 theory determines *w*, which effectively increases the visual cylinder (equation 15) and reduces pursuit success (equation 20). Our linear reduction in pursuit success to zero at *w*>*y*_turb_ (1 *L* s^−1^) was more simplistic than previous approaches, but gave very similar results (e.g., there was a correlation of R^2^ = 95% between equation 20 and Fiksen and MacKenzie’s preferred “Mod4” [83] in the *w* range from 0.1 to 10 mm s^−1^). Note that *in situ* turbulence varies with depth, allowing larvae to influence their exposure by vertical swimming behavior. Since Quirks does not account for such behavior, it makes little sense to address turbulence in even greater detail. For the same reason, light intensity was simplified as either insufficient (nighttime) or sufficient (daytime) for visual feeding, even though it varies continuously with time and depth in the field.

### Known Limitations

Mathematical models of complex biophysical processes always have limitations, and Quirks is no exception. First, several environmental factors that may particularly influence larval fish foraging and growth in estuarine nursery areas (e.g., salinity, oxygen, turbidity), were not included in the model. With respect to our validation dataset, any one of these variables presumably contributed only a small portion of the ∼35% unexplained (and photoperiod-unrelated) variability in published growth rates. Nevertheless, Quirks is free software, and users may extend the model to include additional variables and processes.

#### Stochasticity

Another limitation of Quirks (and many other IBMs) is that highly stochastic processes such as prey encounters are represented by simple averages. On the one hand, this allows for a fully deterministic model, in keeping with the design goal of transparency. On the other hand, this precludes Quirks (in its current form) from modeling stochastic variability in growth or foraging success. Further, the lack of a state variable tracking larval gut contents means that various behavioral and physiological mechanisms related to stochastic foraging success are not represented. For example, modeled larvae cannot fill their guts towards the beginning and then save energy towards the end of a modeled time step. They also cannot benefit from increased metabolic efficiency during times of reduced gut turnover [30], [61], [84], e.g. after dusk.

#### Thermal tolerance

Larval tolerance of high temperatures does not emerge mechanistically from the model and must therefore be defined *a priori* via the *x*_tol_ trait. This causes modeled growth potential to increase with temperature until just below the specified upper limit, which is unrealistic [3], [85]. Instead, unfavorable enzyme kinetics or insufficient aerobic scope for metabolism should cause growth potential to diminish as temperature approaches an ecological limit somewhat below the physiologically lethal temperature. We are not aware of any larval fish model that successfully incorporates such proximate mechanisms. There is a pressing need for additional research into the mechanisms limiting larval fish thermal tolerance, so that models such as Quirks can become more useful for climate research. Until this is accomplished, extra care should be taken in the interpretation of model growth predictions for temperatures that have not been empirically validated.

### Summary and Conclusions

#### North sea fish larvae

The most distinguishing features of young exogenously feeding anchovy, cod, herring (autumn-spawned), and sprat larvae at environmental conditions they typically experience in the North Sea were as follows. Young anchovy larvae were shortest, had the fastest metabolism, required a high prey concentration, and had the potential to grow fast. Young cod larvae were heavy and had the slowest metabolism, benefited from large prey, required a high prey concentration, and achieved the lowest growth potential. Young herring larvae were both long and heavy, benefited from large prey, had a low prey requirement, and could grow fast. Young sprat larvae were lightest and required the lowest prey concentration despite being limited to feeding on the smallest prey.

#### Quirks

We found that Quirks could mechanistically explain the majority of variability among growth measured by numerous approaches for larvae of four species, from <30 to >300 µg in dry mass, at temperatures from <3 to >24°C and at photoperiods from <10 h to continuous daylight. To the best of our knowledge, this level of model skill is unprecedented for a diverse set of fish larvae and environments. By releasing Quirks as free software (Source Code S1, https://sourceforge.net/projects/larvalfishquirks/), we hope to encourage others to build on our efforts.

## Supporting Information

Source Code S1 Quirks_1.00.R.doc.
(DOC)
