# Supplementary material for: A Day in the Life of Fish Larvae: Modeling Foraging and Growth Using Quirks
Source: PLoS One. 2014 Jun 5;9(6):e98205. doi: 10.1371/journal.pone.0098205 (PMC4046954; doi:10.1371/journal.pone.0098205)
Supplement: Source Code S1 — Quirks_1.00.R.doc. (DOC) [file pone.0098205.s001.doc]

#

# Quirks version 1.00 (2013-10-14)

# Copyright (c) 2013, Klaus B. Huebert

# All rights reserved.

#

# Redistribution and use in source and binary forms, with or without

# modification, are permitted provided that the following conditions are met:

#

# 1. Redistributions of source code must retain the above copyright notice, this

# list of conditions and the following disclaimer.

# 2. Redistributions in binary form must reproduce the above copyright notice,

# this list of conditions and the following disclaimer in the documentation

# and/or other materials provided with the distribution.

#

# THIS SOFTWARE IS PROVIDED BY THE COPYRIGHT HOLDERS AND CONTRIBUTORS "AS IS" AND

# ANY EXPRESS OR IMPLIED WARRANTIES, INCLUDING, BUT NOT LIMITED TO, THE IMPLIED

# WARRANTIES OF MERCHANTABILITY AND FITNESS FOR A PARTICULAR PURPOSE ARE

# DISCLAIMED. IN NO EVENT SHALL THE COPYRIGHT OWNER OR CONTRIBUTORS BE LIABLE FOR

# ANY DIRECT, INDIRECT, INCIDENTAL, SPECIAL, EXEMPLARY, OR CONSEQUENTIAL DAMAGES

# (INCLUDING, BUT NOT LIMITED TO, PROCUREMENT OF SUBSTITUTE GOODS OR SERVICES;

# LOSS OF USE, DATA, OR PROFITS; OR BUSINESS INTERRUPTION) HOWEVER CAUSED AND

# ON ANY THEORY OF LIABILITY, WHETHER IN CONTRACT, STRICT LIABILITY, OR TORT

# (INCLUDING NEGLIGENCE OR OTHERWISE) ARISING IN ANY WAY OUT OF THE USE OF THIS

# SOFTWARE, EVEN IF ADVISED OF THE POSSIBILITY OF SUCH DAMAGE.

#

### parameterizations for Huebert and Peck (2014) PLOS ONE

anchovy **<-** list**(** name **=** "anchovy" ,

len.min **=** 5.5 , # minimum standard length (mm)

len.max **=** 7.5 , # maximum standard length (mm)

T.act **=** 2 , # cost of foraging activity ("routine respirations")

T.body **=** 0.2258 , # body shape (µg/mm³)

T.det **=** 0.07 , # prey length at 50% detectability (mm)

T.dig **=** 0.025 , # digestion at 10°C ("dry masses"/h)

T.dist **=** 0.5 , # encounter distance ("standard lengths")

T.dQ10 **=** 2.5 , # digestive Q10 (unitless)

T.eff **=** 0.675 , # efficiency of digestion (unitless)

T.hand **=** 1.5 , # handling time (s)

T.ing **=** 0.08050 , # ingestible prey length ("standard lengths")

T.len **=** 5.5 , # initial standard length (mm)

T.res **=** 0.004113 , # routine respiration at 10°C ("dry masses"/h)

T.rQ10 **=** 1.385 , # respiratory Q10 (unitless)

T.swim **=** 0.75 , # swimming speed ("standard lengths"/s)

T.tol **=** 25 , # upper thermal tolerance (°C)

T.turb **=** 1 , # corrigible turbulent velocity ("standard lengths"/s)

T.vis **=** 0.6 **)** # effective visual cylinder radius ("standard lengths")

cod **<-** list**(** name **=** "cod" ,

len.min **=** 7 ,

len.max **=** 9 ,

T.act **=** 2 ,

T.body **=** 1.044 ,

T.det **=** 0.07 ,

T.dig **=** 0.025 ,

T.dist **=** 0.5 ,

T.dQ10 **=** 2.5 ,

T.eff **=** 0.675 ,

T.hand **=** 1.5 ,

T.ing **=** 0.1571 ,

T.len **=** 7 ,

T.res **=** 0.002032 ,

T.rQ10 **=** 2.384 ,

T.swim **=** 0.75 ,

T.tol **=** 15 ,

T.turb **=** 1 ,

T.vis **=** 0.6 **)**

herring **<-** list**(** name **=** "herring" ,

len.min **=** 13 ,

len.max **=** 15 ,

T.act **=** 2 ,

T.body **=** 0.1678 ,

T.det **=** 0.07 ,

T.dig **=** 0.025 ,

T.dist **=** 0.5 ,

T.dQ10 **=** 2.5 ,

T.eff **=** 0.675 ,

T.hand **=** 1.5 ,

T.ing **=** 0.07836 ,

T.len **=** 13 ,

T.res **=** 0.002404 ,

T.rQ10 **=** 1.711 ,

T.swim **=** 0.75 ,

T.tol **=** 20 ,

T.turb **=** 1 ,

T.vis **=** 0.6 **)**

sprat **<-** list**(** name **=** "sprat" ,

len.min **=** 7 ,

len.max **=** 9 ,

T.act **=** 2 ,

T.body **=** 0.06841 ,

T.det **=** 0.07 ,

T.dig **=** 0.025 ,

T.dist **=** 0.5 ,

T.dQ10 **=** 2.5 ,

T.eff **=** 0.675 ,

T.hand **=** 1.5 ,

T.ing **=** 0.03837 ,

T.len **=** 7 ,

T.res **=** 0.002404 ,

T.rQ10 **=** 1.711 ,

T.swim **=** 0.75 ,

T.tol **=** 16 ,

T.turb **=** 1 ,

T.vis **=** 0.6 **)**

prey.lo **=** 0.04 # lower prey length (mm)

prey.bin **=** 0.01 # prey length bin size (mm)

prey.hi **=** 2.00 # upper prey length (mm)

### initialization

prey.len **<-** seq**(**prey.lo**+**prey.bin**/**2,prey.hi**-**prey.bin**/**2,by**=**prey.bin**)** # characteristic lengths for each prey size bin (mm)

init.fish **<-** **function(**name,Temp,photo,biomass**=**1e6,slope**=-**1.2,epsilon**=**1e**-**7**)** # sets up traits and fish/conditions

**{** traits **<<-** eval**(**as.name**(**name**))**

fish **<<-** expand.grid**(** Temp **=** Temp , # temperature (°C)

photo **=** photo , # photoperiod (h daylight)

biomass **=** biomass , # prey (dry) biomass (mg/m³)

slope **=** slope , # normalized prey size spectrum (unitless)

epsilon **=** epsilon **)** # turbulent kinetic energy dissipation (W/kg)

fish**$**len **<<-** traits**$**T.len

fish**$**mass **<<-** DM.from.SL**(**fish**$**len**)** **}**

### prey field

prey.DM **<-** **function(**x**=**prey.len**)** # individual prey dry mass (µg)

8.6*****x**^**2.1

prey.int **<-** **function(**slope,lo**=**prey.lo,hi**=**prey.hi**)** # preyfield integral (µg)

**{** slope **<-** slope**+(**slope**+**1**==**0**)***1e**-**6 # prevents division by zero

prey.DM**(**hi**)^(**slope**+**1**)/(**slope**+**1**)-**prey.DM**(**lo**)^(**slope**+**1**)/(**slope**+**1**)** **}**

prey.BM **<-** **function(**biomass,slope**)** # prey bin biomass (mg/m³), DW

biomass*****prey.int**(**slope,prey.len**-**prey.bin**/**2,prey.len**+**prey.bin**/**2**)/**prey.int**(**slope**)**

prey.conc **<-** **function(**biomass,slope**)** # prey concentration (1/mm³)

1e**-**6*****prey.BM**(**biomass,slope**)/**prey.DM**()**

### foraging

handling **<-** **function()** # handling time, i.e. prey encounter duration (s)

traits**$**T.hand

max.prey **<-** **function(**len**)** # maximum ingestible prey length (mm)

traits**$**T.ing*****len

max.w.turb **<-** **function(**len**)** # maximum turbulent velocity (mm/s)

traits**$**T.turb*****len

u.pred **<-** **function(**len**)** # predator swimming velocity (mm/s)

traits**$**T.swim*****len

v.prey **<-** **function()** # prey swimming velocity (mm/s)

prey.len*****3

w.turb **<-** **function(**len,epsilon**)** # relative predator-prey turbulent velocity (mm/s)

55**/**18*****0.53***(**traits**$**T.dist*****len*****1e6*****epsilon**)^(**1**/**3**)**

V.combined **<-** **function(**len,epsilon**)** # combined velocity (mm/s)

sqrt**(**u.pred**(**len**)^**2**+**v.prey**()^**2**+**w.turb**(**len,epsilon**)^**2**)**

observation **<-** **function()** # probability of detecting nearby prey (unitless)

prey.len**/(**prey.len**+**traits**$**T.det**)**

encounter **<-** **function(**len,biomass,slope,epsilon**)** # perceived encounter rate (1/s)

V.combined**(**len,epsilon**)***pi***(**traits**$**T.vis*****len**)^**2*****prey.conc**(**biomass,slope**)***observation**()**

pursuit **<-** **function(**len,epsilon**)** # probability of successful pursuit (unitless)

pmax.int**(**1**-**w.turb**(**len,epsilon**)/**max.w.turb**(**len**)**,0**)**

capture **<-** **function(**len**)** # probability of successful capture (unitless)

pmax.int**(**1**-**prey.len**/**max.prey**(**len**)**,0**)**

meal **<-** **function(**len,epsilon**)** # expected DM ingested per encounter (µg)

prey.DM**()***pursuit**(**len,epsilon**)***capture**(**len**)**

ranking **<-** **function(**len,epsilon**)** # order of prey bins from most to least favourable (unitless)

order**(**meal**(**len,epsilon**)/**handling**()**,decreasing**=**T**)**

cum.meal **<-** **function(**len,biomass,slope,epsilon**)** # ingestion (without handling) by diet composition (µg/s)

cumsum**((**meal**(**len,epsilon**)***encounter**(**len,biomass,slope,epsilon**))[**ranking**(**len,epsilon**)])**

cum.hand **<-** **function(**len,biomass,slope,epsilon**)** # fraction of time engaged in handling by diet (unitless)

cumsum**((**handling**()***encounter**(**len,biomass,slope,epsilon**))[**ranking**(**len,epsilon**)])**

diets **<-** **function(**len,biomass,slope,epsilon**)** # ingestion (with handling) by diet (µg/s)

cum.meal**(**len,biomass,slope,epsilon**)/(**1**+**cum.hand**(**len,biomass,slope,epsilon**))**

foraging **<-** **function(**len,biomass,slope,epsilon**)** # optimal foraging capacity (µg/h)

max**(**diets**(**len,biomass,slope,epsilon**))***60*****60

foraging **<-** Vectorize**(**foraging,c**(**"len","biomass","slope","epsilon"**))**

### growth

DM.from.SL **<-** **function(**len**)** # dry mass (µg)

traits**$**T.body*****len**^**3

SL.from.DM **<-** **function(**mass**)** # standard length (mm)

**(**mass**/**traits**$**T.body**)^(**1**/**3**)**

digestion **<-** **function(**mass,Temp**)** # digestive capacity, i.e. max gut content * gut evacuation rate (µg/h)

mass*****traits**$**T.dig*****traits**$**T.dQ10**^((**Temp**-**10**)/**10**)**

respiration **<-** **function(**mass,Temp**)** # routine respiration (µg/h)

mass*****traits**$**T.res*****traits**$**T.rQ10**^((**Temp**-**10**)/**10**)**

grow **<-** **function()** # ("dry masses"/d), contact me for an efficient version suitable for large datasets

**{** f.wrapper **<-** **function()** foraging**(**fish**$**len,fish**$**biomass,fish**$**slope,fish**$**epsilon**)** # (µg/h)

d.wrapper **<-** **function()** digestion**(**fish**$**mass,fish**$**Temp**)** # (µg/h)

r.wrapper **<-** **function()** respiration**(**fish**$**mass,fish**$**Temp**)** # (µg/h)

gain **<-** **function()** ifelse**(**light,pmin.int**(**f.wrapper**()**,d.wrapper**())***light*****traits**$**T.eff,0**)** # (µg)

loss **<-** **function()** r.wrapper**()*(**1**+**traits**$**T.act*****light**)** # (µg)

**if(**max**(**fish**$**len**<**traits**$**len.min**))** # enforces minimum length

**{** fish **<<-** fish**[**fish**$**len**>=**traits**$**len.min,**]**

**if(**nrow**(**fish**)==**0**)**

return**()** **}**

**if(**max**(**fish**$**Temp**>**traits**$**T.tol**))** # enforces maximum thermal tolerance

**{** fish **<<-** fish**[**fish**$**Temp**<=**traits**$**T.tol,**]**

**if(**nrow**(**fish**)==**0**)**

return**()** **}**

**for(**Time **in** 1**:**24**)** # (h)

**{** **if(**max**(**fish**$**len**)>**traits**$**len.max**)** # enforces maximum length

**{** fish **<<-** fish**[**fish**$**len**<=**traits**$**len.max,**]**

**if(**nrow**(**fish**)==**0**)**

return**()** **}**

light **<-** 1**-**pmax.int**(**pmin.int**((**24**-**fish**$**photo**)-(**Time**-**1**)**%%24,1**)**,0**)** # daylight (h)

fish**$**mass **<<-** fish**$**mass**+**gain**()-**loss**()**

fish**$**len **<<-** ifelse**(**fish**$**mass**>**DM.from.SL**(**fish**$**len**)**,SL.from.DM**(**fish**$**mass**)**,fish**$**len**)** **}**

return**(**fish**$**mass**/**DM.from.SL**(**traits**$**T.len**)-**1**)** **}**

### example: grow 7-mm cod at 3 temperature x 3 photoperiod x 3 prey x 3 turbulence levels

init.fish**(** name **=** "cod" ,

Temp **=** c**(**8,10,12**)** ,

photo **=** c**(**8,12,16**)** ,

biomass **=** c**(**40,20,10**)** ,

slope **=** **-**1.2 ,

epsilon **=** c**(**1e**-**9,1e**-**7,1e**-**5**)** **)**

fish**$**growth **<-** grow**()**

unlist**(**traits**)**

head**(**fish,18**)**

summary**(**fish**)**
